# Long non-coding RNA in hepatocellular carcinoma: mechanistic insights and therapeutic perspectives

**DOI:** 10.1007/s13402-025-01119-9

**Published:** 2025-11-04

**Authors:** Kexin Yu, Yuanxiang Jin, Yidong Zhou, Qiaoping Xu

**Affiliations:** 1 Fourth Clinical Medical College of Zhejiang Chinese Medical University, Affiliated Hangzhou First People’s Hostipal, Hangzhou, 310053 China; 2 Department of Clinical Pharmacology, Key Laboratory of Clinical Cancer Pharmacology and Toxicology Research of Zhejiang Province, Cancer Center, Affiliated Hangzhou First People’s Hospital, Westlake University School of Medicine, Hangzhou, 310006 China

**Keywords:** Hepatocellular carcinoma, Long non-coding rNAs, Biomarker, Molecular mechanism

## Abstract

Hepatocellular carcinoma (HCC) remains one of the most lethal malignancies worldwide, with its pathogenesis involving complex biological processes such as DNA damage, epigenetic modification and oncogene mutation. Over the past two decades, the role of long non-coding RNAs (lncRNAs) in the occurrence, metastasis and progression of HCC has received increasing attention. As an important noncoding RNA molecule, lncRNAs play a key role in regulating gene expression, affecting RNA transcription and mRNA stability. This review elucidates the potential pathogenic processes of HCC and elaborates on the synthesis mechanisms of the above three non-coding RNAs. It comprehensively summarizes various non-coding RNAs that have been identified as playing key regulatory roles in HCC, as well as how these non-coding RNAs affect disease progression by regulating gene expression and protein functions. For example: lncRNAs such as NEAT1, DSCR8, PNUTS, HULC, and HOTAIR can play different roles in the proliferation, migration, and apoptosis of HCC cells in different ways. lncRNAs such as HClnc1, LINC01343, FAM111A-DT, CERS6–AS1, and TLNC1 significantly affect the progression of HCC by regulating key signaling axes or protein functions, and are closely related to the prognosis of patients. In addition, we also discuss the potential of lncRNAs as therapeutic targets for HCC, such as: lncRNA MIR31HG, CASC2c, and lncRNA AC115619. Furthermore, we also explore the application prospects of lncRNAs as potential biomarkers and therapeutic targets, providing new perspectives and directions for future HCC research.

## Clinical trial number

Not applicable.

## Introduction

Primary liver cancer (PLC) ranks among the most prevalent malignancies globally, with hepatocellular carcinoma (HCC) constituting 75% ~ 85% of all PLC cases, making it the predominant pathological subtype [1]. As a vital metabolic organ, the liver plays a crucial role in maintaining carbohydrate and lipid homeostasis. However, the occurrence of HCC is often accompanied by liver dysfunction, leading to metabolic rearrangements, which have multiple effects on the human body. Epidemiological data reveal a concerning rise in HCC incidence, particularly in the United States, where the age-adjusted rate has surged from 1.5 to 4.9 cases per 100,000 individuals over the past three decades. HCC has microenvironmental features such as low pH,M2 tumor-associated macrophage enrichment, low oxygen, rich blood supply and susceptibility to hematotropic metastasis, high chemokine expression, enzyme overexpression, high redox level, and strong immunosuppression, which not only promotes the progression of the disease, but also seriously affects the clinical effectiveness of traditional therapeutic approaches [2]. Patients with HCC have a variety of treatment options, including liver transplantation, surgical resection, percutaneous radiofrequency ablation, and systemic treatment. For early liver cancer, resection surgery is currently the most effective treatment. Nonetheless, resection does not treat the disease in advanced patients, so finding a method with a better prognosis is necessary [3].

Chronic hepatitis B (HBV) and hepatitis C virus (HCV) infection, alcohol consumption, nonalcoholic fatty liver disease (NAFLD) and aflatoxin B1 intake are all risk factors for HCC, which may increase the incidence of HCC [4]. These factors may induce DNA damage, epigenetic changes and cancer-related mutations, leading to the silencing of tumor suppressors (such as TP53, CDH1, RASSF1) and the activation of oncogenes (such as MYC, VEGFA, MAPK7), which ultimately contribute to the progression of HCC [5]. Early HCC-related studies mainly focused on the protein-coding genes due to their central roles in the regulation of biological processes [6]. However, through in-depth study of ncRNAs, more and more evidence shows that evolutionarily conserved noncoding RNAs (ncRNAs), especially long non-coding RNAs (lncRNAs), play a key role in the proliferation, migration, invasion and apoptosis of HCC cells [7]. LncRNAs are a class of RNA molecules with a transcript length greater than 200 nt and lack the ability to encode proteins. It is estimated that the number of lncRNAs in humans has exceeded 60,000 and is still increasing rapidly. Like mRNA, lncRNAs are also transcribed by polymerase II, most of which have 50 caps, polyadenylation and splicing [8]. LncRNAs regulate gene expression by interacting with DNA, RNA, and proteins. These interactions affect many cellular processes, including cell growth and development, and lead to the proliferation of cancer cells. Recently, lncRNAs abnormalities have been shown to have tumor suppressor or carcinogenic effects. For example, a new differentially expressed lncRNA RP11-85G21.1 can promote the proliferation or migration of HCC cells by targeting miR-324-5p. This suggests that it may be used as a new therapeutic target and prognostic marker for HCC. In addition, lncRNA H19 (an endogenous non-coding single-stranded RNA) can affect the proliferation, apoptosis, invasion and metastasis of HCC cells through its epigenetic modification, drug resistance and regulation of its downstream pathways [9]. This indicates that lncRNA H19 plays an oncogene role in the occurrence and progression of HCC. These important roles and unique properties of lncRNAs indicate their potential clinical value in the diagnosis and treatment of HCC. This review aims to summarize the role of lncRNA in HCC and its prospective application in medicine. In addition, we also discussed the diagnostic and therapeutic potential of lncRNAs in HCC.

## Advances in the immune microenvironment and lncRNAs in HCC

### Biological functions of lncRNAs in HCC

#### Overview of lncRNAs

LncRNAs play critical roles in numerous fundamental biological processes, including circadian regulation. Intriguingly, many lncRNAs contain small open reading frames (smORFs) capable of encoding micro peptides approximately 100 amino acids in length. These micro peptides are distinct from the longer functional proteins (often exceeding 400 amino acids) encoded by mRNAs. LncRNAs-encoded micro peptides have been implicated in regulating a variety of life processes, including transcription and regulation of mRNAs. Furthermore, recent studies have revealed that lncRNAs regulate gene expression through multiple mechanisms, including epigenetic modification and interaction with microRNAs (miRNAs) and proteins. They may also function as miRNA precursors or pseudogenes to regulate gene expression at the transcriptional and post-transcriptional levels [10]. In the context of liver cancer, lncRNAs represent a recently discovered class of molecules with significant regulatory roles. And they are involved in the pathogenesis and development of liver fibrosis by regulating signaling pathways including transforming growth factor-β pathway, phosphatidylinositol 3-kinase/protein kinase B pathway, and Wnt/β-catenin pathway [11]. Additionally, dysregulation of lncRNAs has been increasingly associated with various liver diseases, including HCC [12].

LncRNAs can be classified based on their genomic locations relative to protein-coding genes into the following categories: sense lncRNA, antisense lncRNA, bidirectional lncRNA, intron lncRNA, intergenic lncRNA and enhancer lncRNA. Besides, according to whether the expression of lncRNAs promote tumorigenesis and development, lncRNAs can be divided into oncogenes and tumor suppressor genes. In general, overexpressed lncRNAs promote tumor development, so it is classified as an oncogene. On the contrary, down-regulated lncRNAs inhibit tumor development and are considered as a tumor suppressor gene [13]. Furthermore, based on their mechanisms of regulating target gene expression - through either cis- or trans-regulation [8] - lncRNAs can also be divided into cis-acting and trans-acting types.

**Fig. 1:**
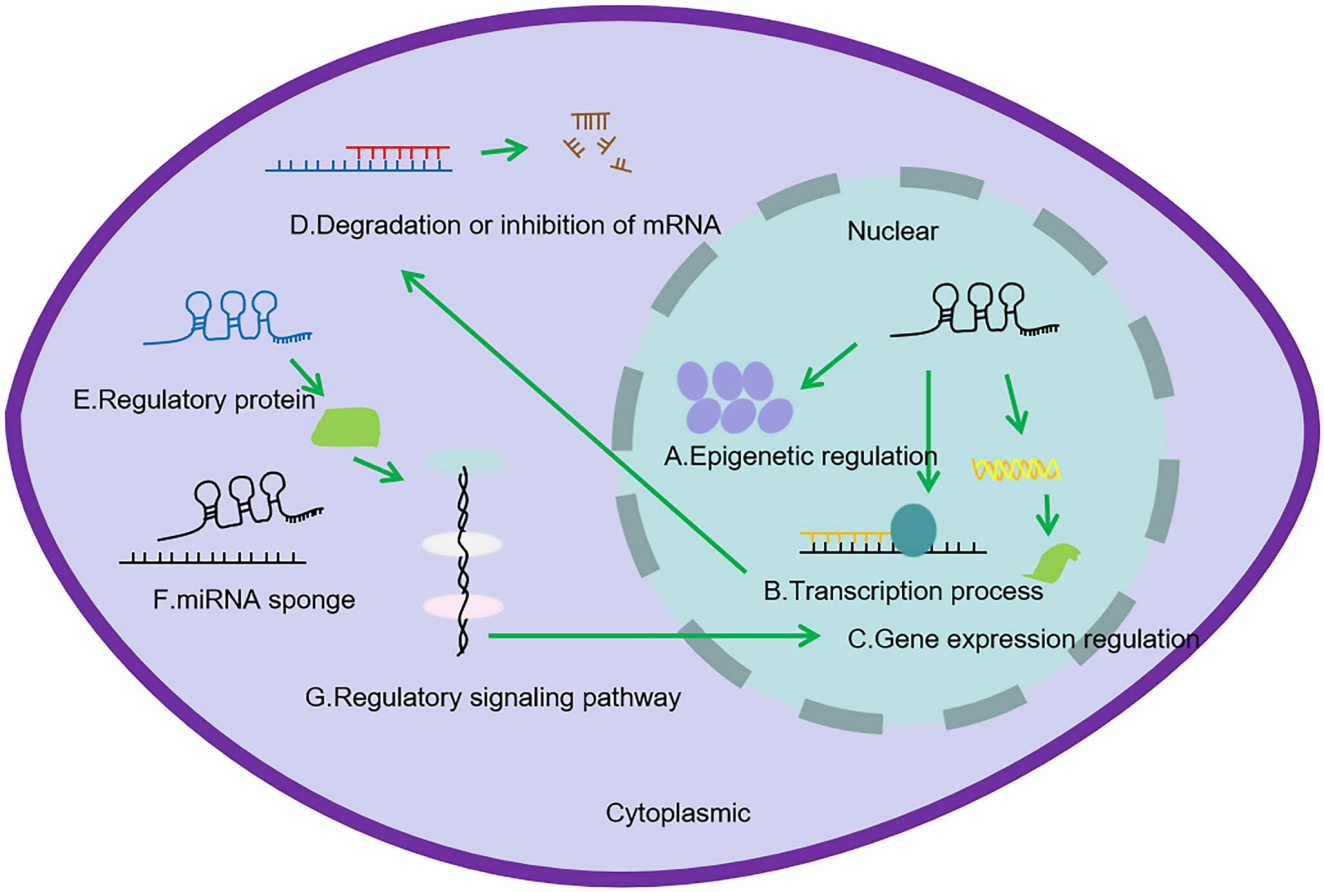
Regulatory mechanisms of lncRNAs in the nucleus and cytoplasm. (**A**) In the nucleus, lncRNA promotes or inhibits gene expression by recruiting regulatory molecules. (**B**) by recruiting regulatory molecules to messenger RNA to regulate messenger RNA processing, (**C**) by regulating histones to promote or inhibit gene expression. (**D**) In the cytoplasm, lncRNAs regulate mRNA stability by directly binding to mRNA. (**E**) LncRNAs interact with proteins to regulate signal cascades. (**F**) LncRNAs act as miRNA sponges to competitively bind miRNA regulation, thereby regulating signal pathways

In addition to high tissue specificity and spatial and temporal specificity, lncRNAs also show a variety of characteristic functions. The function of lncRNAs is largely reflected in their subcellular localization [14]. Studies have shown that most lncRNAs are located in the nucleus [15], but some lncRNAs also play a role in the cytoplasm [16]. Nuclear lncRNAs usually regulate nuclear processes such as RNA transcription, post-transcriptional gene expression and chromatin organization. In contrast, cytoplasmic lncRNA regulates cytokine sponging, cell signaling, mRNA transport, stability and translation, as well as protein stability, post-translational modification and function [17]. LncRNAs can act on different types of biomolecules, which is significant for the proliferation and apoptosis, invasion and migration [14], and drug resistance of HCC cells (Fig. 1). Therefore, understanding the functional mechanism of lncRNAs expression changes may have clinical significance for the diagnosis, treatment and prognosis of HCC. The potential molecular mechanisms and biological effects of lncRNAs have been extensively studied. For example, by down-regulating miRNA-15b expression, lncRNA H19 stimulates the CDC42/PAK1 axis and increases the proliferation rate of HCC cells [18]. In addition, lncRNA -p21 is a hypoxia-responsive lncRNA that forms a positive feedback ring with HIF-1α to drive glycolysis and promote the growth of tumors [19]. Overexpression of linc-RoR（Long intergenic non-coding RNA-ROR） in the extracellular tumor environment during hypoxia in HCC cells has been shown to act as a miR sponge for tumor suppressor miR-145, thereby allowing cancer cells to self-renew. The up-regulation of miR-145 downstream targets p70S6K1, PDK1 and HIF-1α was induced by linc-RoR, resulting in accelerated cell proliferation [20, 21].

Due to their different functions, lncRNAs not only play an important role in a wide range of biological processes, but also play a key role in the diagnosis and prognosis of various cancers, including liver cancer. Importantly, lncRNAs can control the expression of multiple mRNA targets in different cancer-related pathways, so clinical development of anticancer drugs based on the mechanism of lncRNAs have begun [7].

### LncRNAs profiles in HCC

LncRNAs are closely related to the occurrence and progression of HCC, especially by regulating the formation of liver CSC. Studies have found that lncRNAs play a key role in driving CSC self-renewal and tumor proliferation by activating the Wnt/β-catenin pathway [22]. Elucidating the mechanisms by which lncRNAs contribute to HCC pathogenesis may facilitate the development of novel therapeutic strategies and prognostic biomarkers. However, the detailed mechanism remains to be fully elucidated. Therefore, this review summarizes the research progress of lncRNAs in HCC and its potential mechanism of action, in order to promote the understanding of these mechanisms and provide a reference for the future development of lncRNA-based treatment of HCC [22].

LncRNAs were originally thought to be the ‘noise’ of genomic transcription. In 1984, the first eukaryotic lncRNA-H19 was found in mice, with a length of 2.3 kb, and was highly expressed during embryonic development. Since then, people ‘s research interest in lncRNAs have increased significantly, and lncRNAs have begun to attract widespread attention. For example, a study has shown that linc01134 can accelerate HCC progression by down-regulating structure-specific recognition protein 1 (SSRP1). Its prognostic value in HCC and five other up-regulated lncRNAs have been recorded [23, 24]. In addition, experiments have confirmed that more than 70 cases of MEG3 are significantly down-regulated in human HCC and found that low levels of MEG3 are associated with adverse clinical reactions of HCC, resulting in reduced overall survival and recurrence-free survival [25].

In recent years, a large number of lncRNAs have been identified, and a number of studies have investigated the role of lncRNAs in HCC. However, they have not yet carried out clinical practice, and the process of transforming preclinical results into clinical results is difficult or even difficult to achieve. Therefore, the feasibility and safety of ncRNA as a clinical therapeutic target remains to be determined [26].

## Dysregulation and molecular mechanisms of lncRNAs in HCC

### The role of lncRNAs in the proliferation and apoptosis of HCC

The role of lncRNAs in the proliferation and apoptosis of HCC. More and more evidences show that lncRNAs act as a positive or negative regulator of coding genes in the progression of HCC [27, 28]. In addition, lncRNAs can promote the transformation process by interacting with DNA, RNA or protein [29]. LncRNAs can also be used as a competitive endogenous RNA, and its stability is regulated by miRNA, but lncRNAs can isolate specific miRNA from its target gene, thereby inhibiting miRNA-mediated function [30, 31]. This section mainly summarizes several lncRNAs related to the proliferation and apoptosis of HCC.

LncRNA ASH1L-AS1 has the potential to encode a microprotein, APPLE, which is stably expressed in HCC cells and continuously upregulated in tumor tissues. Functionally, APPLE promotes Extracellular signal-regulated kinases 1 and 2 (ERK1/2) phosphorylation, activates The Mitogen-Activated Protein Kinase (MAPK) signaling, and enhances HCC cell proliferation, migration, invasion, and tumor growth effects, which are reversed by APPLE knockdown or ERK1/2 inhibition [32]. LncRNA NEAT1 plays an important role in the occurrence of HCC. It has been found that the up-regulation of lncRNA NEAT1 can increase the expression of Bcl-2 and EGFR, thus promoting the proliferation and invasion of tumor cells [33]. Bcl-2 is a key regulatory gene of the anti-apoptotic protein family, and its apoptosis-inhibiting gene is closely related to its anti-apoptosis [34], while the overexpression of EGFR is involved in the occurrence and development of malignant tumors [34]. Studies have confirmed that transfection of lncRNA NEAT1 small interfering RNA (siRNA) to hepatoma cell HepG2, down-regulation of lncRNA NEAT1 expression, can inhibit the expression of EGFR and Bcl-2, thereby inhibiting tumor cell proliferation and invasion. MiR-22-3p can regulate the expression of ARPC5 by binding to ARPC5 and ARPC5 can promote the occurrence and development of HCC [35]. LncRNA _ DSCR8 is the upstream lncRNA of miR-22-3p. Therefore, lncRNA_ DSCR8 can promote the occurrence of HCC by regulating ARPC5 through sponge miR-22-3p. Overall, DSCR8 is overexpressed in HCC cells and plays an important role in regulating the proliferation and apoptosis of HCC cells.

In HCC, lncRNA PNUTS promotes HCC cell proliferation and dispersion by binding to ZEB1 [29]. In addition, lncRNA02273 can significantly promote the movement, invasion and proliferation of Hep3B and MHCC97 cells, while reducing apoptosis in HCC [36]. In addition, studies have shown that up-regulation of lncRNA HULC and HOTAIR reduces apoptosis and chemosensitivity, while accelerating the proliferation, invasion and metastasis of HCC cells [29]. In human Huh7 and SMMC-7721 hepatocytes cell lines, lncRNA KCNQ1OT1 can promote S1PR1 expression by sponging miR-149, which can downregulate S1PR1expression by targeting the 30-UTR of S1PR1 mRNA, which then facilitate the progress of HCC [37].

**Fig. 2:**
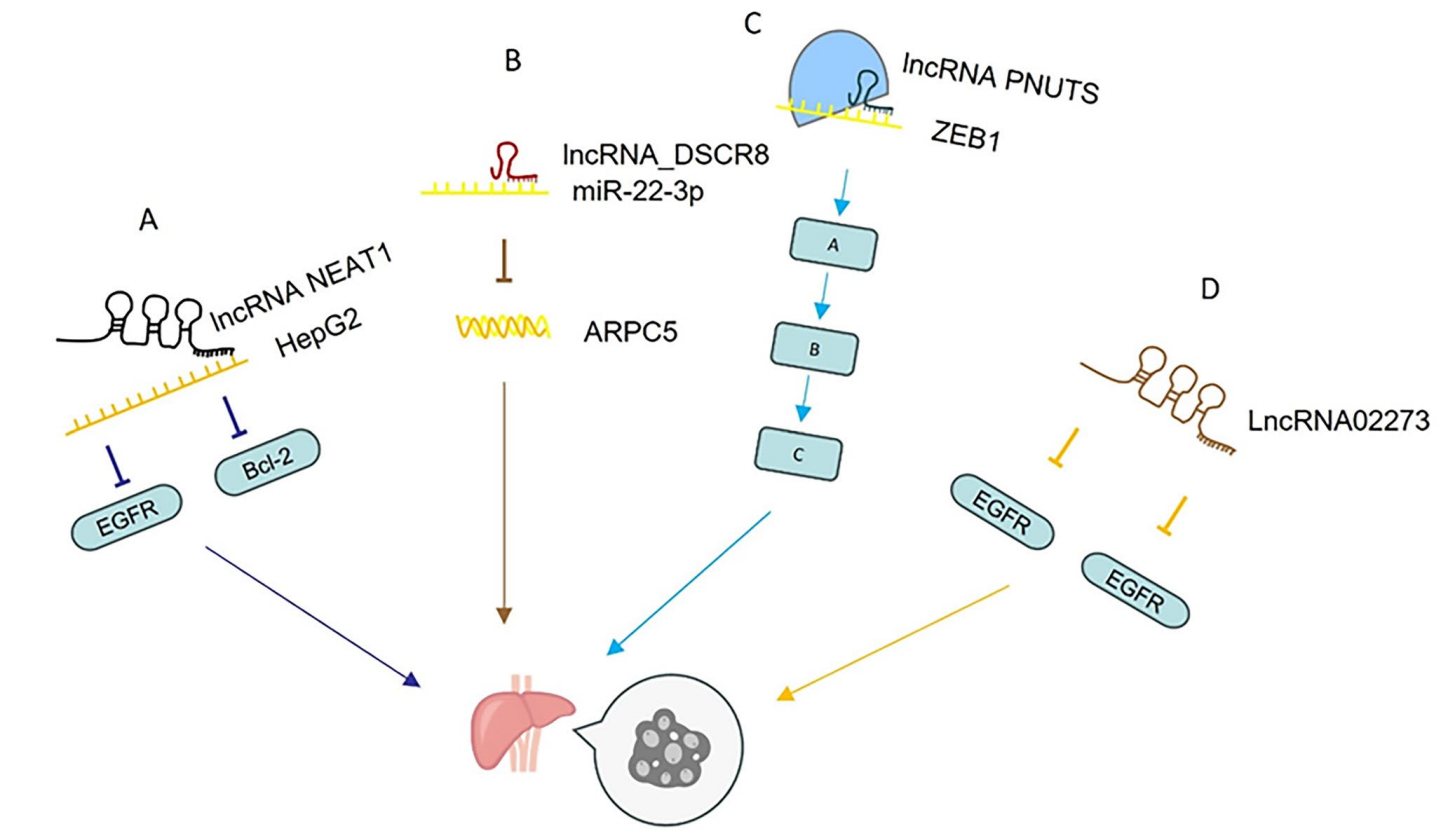
Mechanism of lncRNAs regulating proliferation and apoptosis of HCC. (**A**) The up-regulation of lncRNA NEAT1 can increase the expression of Bcl-2 and EGFR. (**B**) lncRNA_DSCR8 regulates ARPC5 through sponge miR-22-3p. (**C**) lncRNA PNUTS promote the proliferation and dispersion of HCC cells by binding to ZEB1. (**D**) LncRNA02273 promoted the proliferation of Hep3B and MHCC97 cells

In summary, lncRNA ASH1L-AS1, NEAT1, DSCR8, PNUTS, HULC, and HOTAIR can play different roles in the proliferation, migration, and apoptosis of HCC cells in different ways. In subsequent studies, they can be developed as molecular tools suitable for HCC treatment [31]. The role and mechanism of lncRNAs in the proliferation and apoptosis of HCC are shown in the table (Fig. 2).

### Roles of lncRNAs in migration, invasion and EMT of HCC

NcRNAs, which include microRNAs (miRNAs), lncRNAs, circular RNAs (circRNAs), and small vault RNAs (vtRNAs), modulate a wide array of biological functions [1]. LncRNAs have a critical role in the development and progression of HCC.HCC-related lncRNAs have been demonstrated to exhibit abnormal expression and contribute to transformation process (such as proliferation, apoptosis, accelerated vascular formation, and gain of invasive potential) through their interaction with DNA, RNA or proteins. LncRNAs can bind mRNAs to release their target mRNA and enable its translation. These lncRNA-miRNA networks regulate cancer cell expression and so its proliferation, apoptosis, invasion, metastasis, angiogenesis, epithelial-mesenchymal transition (EMT), drug resistance and autophagy [38]. Previous academic studies have highlighted the indispensable roles of tumor-associated macrophages (TAMs) and exosomes in the progression of HCC. However, the subtle molecular mechanisms by which tumor-derived exosomal lncRNAs interact with TAMs to regulate macrophage polarization and HCC proliferation remain largely unclear. Tumor-derived exosomal SLC12A2-DT (divergent transcript of SLC12A2) promotes HCC progression by regulating the interaction between HCC cells and TAMs via the Wnt/GSK3β/β-catenin signaling pathway [39].

The reprogramming of lipid metabolism serves an important role in occurrence and development of HCC. Fatty acid hydroxylase domain containing 2 (FAXDC2) is a hydroxylase involved in the synthesis of cholesterol and sphingomyelin and downregulated in various types of cancer [40]. However, one experiment showed that Silencing FAT1 could inhibit the proliferation, migration, invasion and EMT of HCC cells. miR-223-3p targeted down-regulated the expression of FAT1, and inhibited the proliferation, migration, invasion and EMT of HCC cells by targeting FAT1. MiR-223-3p regulates the occurrence and development of HCC cells by targeted down-regulating the expression of FAT1 [41]. Xeroderma pigmentosum group D (XPD) inhibits cell growth of HCC. XPD and miR-29a-3p were downregulated, and MIAT and COL4A1 were upregulated in tumor tissues of HCC patients. The same phenomenon was observed in HCC cell lines. The promotion of the malignant phenotype of HCC cells mediated by MIAT overexpression was reversed by COL4A1 deficiency. Mechanistically, MIAT enhanced the expression of COL4A1 by sponging miR-29a-3p. In conclusion, XPD recruits P53 to regulate the MIAT/miR-29a-3p/COL4A1 axis, which contributes to the inhibition of migration, invasion, EMT, and metastasis of HCC [42]. Meanwhile, a study found that YAP expression increases significantly in HCC and it may be involved in the occurrence and development of HCC. YAP expression is an independent risk factor affecting the overall survival of HCC patients [43]. Currently there are more than 100 kinds of heat shock protein (HSP) HSP90 substrate proteins, accumulating evidences showed that many of HSP90 is closely related to tumor invasion and metastasis [44]. Carcinoma HepG2 cell transfected high expression of HSP90can promote the transformation of EMT, improve the expression of Vimentin, reduce the expression of E-cadherin, and inhibit apoptosis of cancer stem cells, which improve the invasive ability of cancer of the liver cells. In conclusion, the expression of HSP is closely related to the occurrence, development and invasion of cancer of the liver tissue [44].

A study of HCC proved that: CYTOR was upregulated and miR-125a-5p was downregulated in HCC cells. CYTOR silencing inhibited cell proliferation and promoted cell apoptosis in HepG2 and SMMC-7721 cells. miR-125a-5p was sponged and negatively regulated by CYTOR, and HAX-1 was directly targeted and negatively modulated by miR-125a-5p [45]. Overexpression of miR-125a-5p enhanced the repressive effects of CYTOR knockdown on HCC cells, and knockdown of HAX-1 enhanced the inhibitory effects of miR-125a-5p mimics on HCC cells. CYTOR silencing facilitates HCC cell apoptosis in vitro via the miR-125a-5p/HAX-1 axis [46]. A series of follow-­up studies have shown that SNHG4 promoted the progression and malignancy of HCC through upregulating CREB5 via sponging miR­211­5p.the above findings suggest that SNHG4 promotes HCC malignancy through the SNHG4/miR­211­5p/CREB5 axis [47]. Small nucleolar RNA host gene 1 (SNHG1) is an important member of the SNHG family. SNHG1 expression is consistently increased in various HCC-related processes, such as cell proliferation, apoptosis, angiogenesis, migration, invasion, and therapy resistance. Higher SNHG1 expression levels predict worse prognosis by positively correlating with clinicopathological features, including larger tumor size, poor differentiation, and advanced stage of HCC patients [48]. DACT3–AS1 was verified to promote metastasis in HCC. Mechanistically, DACT3–AS1 promotes the interaction between HDAC2 and FOXA3 to stimulate FOXA3 deacetylation, which consequently downregulates the FOXA3 protein. Furthermore, FOXA3 serves as a transcription factor that can bind to the PKM2 promoter region, thus hindering PKM2 expression. To summarize, this study uncovered that HIF-1α-induced DACT3–AS1 promotes metastasis in HCC and can upregulate PKM2 via the HDAC2/FOXA3 pathway in HCC cells [49].

Cancer metastasis greatly increases the mortality of HCC by cell migration and changing the arrangement of EMT cells. A large number of lncRNAs have been found to play an important regulatory role in the migration and invasion of HCC. Nuclear lncRNAs can not only recruit other components to regulate mRNA, but also induce methylation to regulate gene transcription and the binding of transcription factors to promoters. Studies have found that H19 is a cancer-promoting factor in HCC cells. Its expression level in HCC tissues is significantly higher than that in normal tissues. Knockout of H19 gene will greatly reduce the proliferation and invasion of HCC cells. H19 can inhibit HCC cell migration through Hnrnpu/PCAF/RNA Pol-II, and can also activate the miR-200 family by increasing histone acetylation, thereby inhibiting HCC metastasis [50]. In addition, H19, as a downstream target of AKT/GSK3T/GSK25A signaling pathway, plays an important role in the invasion and metastasis of HCC [50]. H19 can also activate the CDC42/PAK1 signaling pathway in HCC by targeting miR-15b, thereby promoting cell proliferation, invasion and migration [50]. By reducing the expression of HOTTIP, it can significantly inhibit the distant metastasis of HCC. Through further studies, we found that miRNA-125b acts as a post-transcriptional downstream target of HOTTIP in HCC cells [51]. Overexpression of HOTTIP may lead to the inhibition of miRNA-125b expression, thereby enhancing the invasion and metastasis of HCC [50]. Therefore, HOTTIP can regulate the invasion and metastasis of HCC by regulating the expression of miRNA-125b. Other than that, lncRNA CRNDE is increased in HCC tissues and HCC cells. Knockout of CRNDE can inhibit the growth and metastasis of HCC tumors, inhibit EMT and Wnt/β-catenin pathway in HCC cells [52]. LncRNA AGAP2–AS1 is increased in HCC tissues and HCC cells. Overexpression of AGAP2–AS1 is beneficial to cell proliferation, invasion, migration and EMT of HCC [53].

**Fig. 3:**
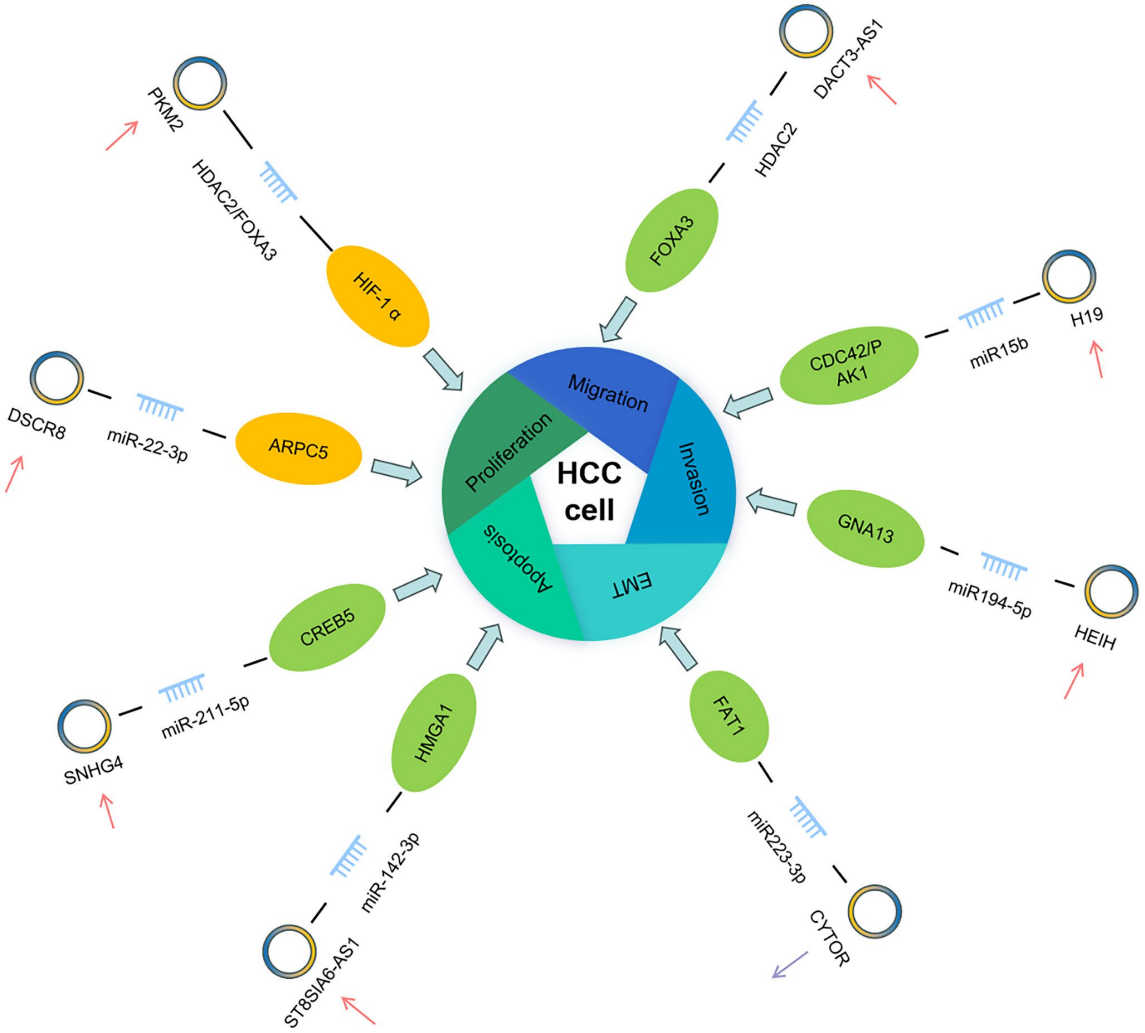
LncRNAs such as H19, DACT3–AS1, SNHG4, and ST8SIA6–AS1 bind to specific miRNAs and modulate the expression of downstream targets—including ARPC5, CREB5, HMGA1, CDC42/P-AKT1, and GNA13. Through these interactions, lncRNAs exert regulatory control over critical cellular processes in HCC, such as proliferation, migration, invasion, apoptosis, and EMT

All things considered, H19, lncRNA HOTTIP, lncRNA CRNDE and lncRNA AGAP2–AS1 are potential therapeutic targets for improving HCC by inhibiting migration, invasion and EMT. The study of the relationship between lncRNAs and HCC progression provides new ideas for the treatment of HCC (Fig. 3).

Related studies have found that lncRNAs are involved in the regulation of cancer cell stress, including metabolic stress, oxidative stress and genotoxic stress [54]. LncRNAs in cancer related metabolic stress. An early study showed that liver-rich PPARα target lncRNA Gm15441 reduced liver inflammation and metabolic stress by inhibiting Txnip-mediated NLRP3 inflammasome pathway [55]. Another study found that PPARα-regulated liver lncRNA G23Riki is a potential metabolic regulator that is specifically up-regulated in the liver in response to PPARα activation. In this study, fasting induced lipid accumulation in the liver of G23Rik-/-mice compared to fasting wild-type mice. The levels of TG, CHOL and NEFA in liver were significantly increased, which was related to the induction of Cd36, an important lipid metabolism-related gene [56]. LncRNAs in cancer‑related oxidative stress. Studies have found that lncRNAs may regulate oxidative stress-related mRNA expression by acting as a competitive endogenous RNA (ceRNA) of miRNA in HCC [57]. Peroxisome proliferator-activated receptor γ coactivator 1α (PGC-1α) is an important transcriptional coactivator that regulates mitochondrial biogenesis and mtDNA replication [58, 59]. In addition, MALAT1 regulates apoptosis and oxidative stress of human lens epithelial cells through p38MAPK pathway [60]. The expression level of Keap1 is inhibited by MALAT1, which will lead to the activation and stability of Nrf2 protein, thereby reducing OS-mediated damage and lipid peroxidation in H2O2-treated HUVEC [61]. LncRNAs in cancer related genotoxic stress. Related studies have found that LUCAT1, AL031985.3 and AC015908.3 these three lncRNAs related to bile acid and bile salt metabolism can independently predict the prognosis of HCC patients and can be used as potential predictors of treatment response [62]. Studies have shown that ZFPM2–AS1, as a ceRNA, promotes DNA damage repair and HCC proliferation through miR-3065-5p/XRCC4 [63]. ZCCHC4 inhibits DNA damage-induced apoptosis of HCC cells by interacting with lncRNA AL133467.2 and hindering its apoptosis. Moreover, the knockout of ZCCHC4 promotes the interaction between AL133467.2 and γH2AX, thereby enhancing the chemosensitivity of HCC cells [64].

In general, lncRNAs play multiple roles in regulating the stress response of cancer cells. For example, lncRNAs such as Gm15441 and G23Riki are involved in regulating liver metabolic stress, affecting lipid accumulation and inflammatory response. And mALAT1 reduces cell damage by regulating oxidative stress pathway. In genotoxic stress, some lncRNAs, as ceRNAs, are involved in DNA damage repair and apoptosis regulation, thus affecting the progression of HCC and chemotherapy sensitivity. These studies reveal the key functions of lncRNAs in cancer development and provide new potential targets and biomarkers for cancer treatment.

### LncRNAs in drug resistance of HCC

HCC is generally sensitive to chemotherapy, but drug resistance remains a major obstacle to achieving favorable patient outcomes. LncRNAs can not only recruit epigenetic regulatory complexes, but also act as a sponge for miRNAs after gene transcription, regulating downstream signal transduction cascades. Moreover, lncRNAs have been shown to influence drug resistance in HCC by regulating gene transcription.

Studies have revealed that CAHM, a key lncRNA, is highly expressed in sorafenib resistant cell lines. It may regulate tumor-related biological processes by regulating AKT phosphorylation or dephosphorylation, and may be involved in apoptosis. Additionally, sorafenib affects the sensitivity of HCC patients to drug therapy by affecting the expression of metallothionein (MT) −1 G [65]. Relevant studies have confirmed that lncRNAs can play an important role in drug resistance of HCC through the MIR124-3P.1-FOXO3a axis. Specifically, mir-124-3p.1 significantly enhances the efficacy of sorafenib through its dual interaction with FOXO3a [65]. These findings suggest that lncRNA CAHM may represent a promising therapeutic target for overcoming drug resistance in HCC.

LncRNAs can regulate drug sensitivity of HCC in different ways. LncRNA DUXAP8, which is highly expressed in HCC and associated with poor prognosis, reduces HCC sensitivity to sorafenib induced iron death by acting on SLC7A11, a subunit of the amino acid reverse transporter system xc-, leading to sorafenib resistance [66]. Therefore, combining sorafenib with DUXAP8 silencing has the potential to overcome its resistance and thus improve treatment outcomes in patients with advanced HCC [66]. In addition, lnc SNHG1 activates the Akt signaling pathway by enhancing the transcription of SLC3A2 (solute carrier family 3 member 2). The expression level of SLC3A2 was significantly increased in HCC cells. Knockdown of SLC3A2 enhanced the effects of sorafenib in inhibiting cell viability and inducing apoptosis, and abolished sorafenib-induced Akt activation. Interestingly, SNHG1 was positively regulated by miR-21 in HCC, which may be related to sorafenib-induced translocation of miR-21 to the nucleus [67]. As an m6A-modified lncRNA, KIF9–AS1 promotes the stem cell properties and sorafenib resistance of HCC by promoting USP1-mediated deubiquitination of short stature homeobox 2 (SHOX2). This discovery provides a new potential target for clinical treatment and is expected to improve the prognosis of HCC patients [68].

In conclusion, this study partially elucidates some relationships between lncRNAs and HCC drug resistance, providing a promising method for predicting the chemotherapy response of HCC. LncRNA CAHM, lncRNA DUXAP8, lncRNA SNHG1, and lncRNA KIF9–AS1 provide new ideas for improving treatment resistance in patients with HCC.

### LncRNAs in radio-sensitivity of HCC

Radiotherapy plays an irreplaceable role in controlling localized lesions and improving overall survival in HCC. It suppresses malignant phenotypes such as tumor growth, invasion and migration by modulating tumor metabolism, thereby inhibiting cancer initiation and progression [69]. Emerging evidence indicates that lncRNAs significantly influence the radiosensitivity of tumor cells. For instance, HIF-2α acts as a transcriptional activator of NEAT1 and promotes EMT. Additionally, the tumor suppressor p53 induces NEAT1 expression and facilitates paraspeckle formation [70]. This process enhances chemosensitivity and mitigates DNA damage accumulation during replication [70]. Furthermore, the direct binding target of BCLF1 can induce the expression of NEAT1, and BCLF1 can induce the proliferation and invasion of HCC cells by promoting the expression of NEAT1 [70]. Therefore, NEAT1 may represent a novel therapeutic target in HCC. Moreover, lncRNA KCNQ1OT1 can reduce the apoptosis and radiosensitivity of human Huh7 and Hep3B liver cell lines by interacting with miR-146a-5p in HCC [71].

In conclusion, NEAT1, p53 and lncRNA KCNQ1OT1 are associated with radiosensitivity of HCC. As the understanding of the mechanisms underlying lncRNA-mediated regulation of radiotherapy sensitivity deepens, targeting lncRNAs may offer a promising strategy to enhance the anti-tumor efficacy of radiotherapy in HCC patients.

### LncRNAs as diagnostic and prognostic biomarkers for HCC

Related survival analysis showed that lncRNAs (AC005332.5, ELF3–AS1 and LINC00665) were highly correlated with the overall survival prognosis of HCC. In addition, multivariate Cox risk analysis showed that lncRNA characteristics were statistically significant and could predict clinical outcomes independently of verifying other clinical variables of patients. Therefore, the three lncRNA features of AC005332.5, ELF3–AS1 and LINC00665 may be used as excellent diagnostic biomarkers for HBV-related HCC and have potential prognostic significance for HBV-related HCC patients [72]. In addition, related studies have shown that the up-regulation of STMN1 can activate YAP1 signal transduction to promote the occurrence and development of HCC, so STMN1 can be used as a marker for HCC diagnosis. Moreover, studies have found that the up-regulated expression of LINC00152 and UCA1 is associated with HCC, indicating that they can be used as non-invasive biomarkers for HCC [73]. Compared with LO2 (normal liver cells), PCAT6 is abnormally highly expressed in HCC cell lines (Hep G2 and Hep3B). When its expression is inhibited, cell proliferation and migration ability are reduced. Therefore, PCAT6 is likely to be a potential biomarker for predicting the prognosis of HCC [74]. HULC is a carcinogenic lncRNA that has been identified as a highly up-regulated lncRNA in human liver tumors. In vitro, it can promote the occurrence and development of HCC. At the mechanism level, HULC can increase the level of LC3II in a NF-κB-dependent manner, promote the phosphorylation of p65 and IκBκB, and enhance autophagy. The down-regulation of HULC is also related to the impaired in situ growth of HCC in vivo [75]. The link between HULC and autophagy may play a role in disease progression. These results indicate that HULC can provide value as a prognostic biomarker and promoter for HCC development. In addition, studies have shown that the levels of HOTAIR and ICR lncRNA in serum after surgery are significantly reduced. Therefore, serum HOTAIR and ICR lncRNA levels are potential prognostic markers for HCC. The up-regulation of HOTAIR and ICR may contribute to the early diagnosis of HCC and indicate poor prognosis. Compared with other tissues and fetal liver, Top35 LNDH is preferentially expressed in adult healthy liver and is induced in well-differentiated HepaRG cells. It is worth noting that their knockdown affects the expression of other liver-specific genes. Finally, the expression of Top35 LNDH was positively correlated with the degree of tumor differentiation, and more importantly, it was related to the better prognosis of patients [76].

A number of studies have confirmed that a variety of lncRNAs such as AC005332.5, ELF3–AS1, LINC00665, PCAT6, HULC, HOTAIR and LINC00152 are abnormally expressed in HCC, which is closely related to the occurrence, development and prognosis of tumors. These lncRNAs can affect the occurrence and development of cancer by regulating autophagy, activating signaling pathways or acting as ceRNAs. Among them, specific lncRNAs such as Top35 LNDH are related to the degree of tumor differentiation and can be used as potential biomarkers for early diagnosis and prognosis evaluation of HCC.

### LncRNAs as potential therapeutic targets for HCC

The molecular complexity, genetic and epigenetic changes, and signaling pathway disorders of HCC promote personalized treatment strategies based on molecular spectrum analysis. Epigenetic regulation, including DNA methylation, histone modification and ncRNA, is a key layer affecting the development of HCC. LncRNAs have attracted attention due to their multiple roles in gene regulation and their potential as tools for cancer diagnosis and treatment. Recent evidence has shown that lncRNAs play a crucial role in the occurrence and progression of HCC [77]. HClnc1 is involved in the new epigenetic mechanism of HCC tumorigenesis and pyruvate kinase (PKM2) regulation. HClnc1 interacts with PKM2 to prevent its degradation, thereby promoting aerobic glycolysis and PKM2–STAT3 signaling. It can be seen that HClnc1 is not only a more accurate prognostic indicator of HCC, but also a potential therapeutic target for HCC treatment. According to the analysis of RNA-seq and public data of liver cancer patients, UPF1 is an HCC inhibitor that plays a role by regulating the lncRNA-HEIH/miR-194-5p/GNA13 axis. The deletion of UPF1 up-regulated the expression of lncRNA-HEIH, and the expression of lncRNA-HEIH was negatively correlated with the expression of miR-194-5p. The overexpression of lncRNA-HEIH inhibited the target of miR-194-5p. In addition, the expression of GNA13 was also negatively correlated with the expression of miR-194-5p. When the miR-194-5p target was inhibited, the level of GNA13 was increased, thereby promoting HCC proliferation. Therefore, UPF1 may be a suitable target for HCC treatment [78].

Studies have found that LINC01343 is up-regulated in HCC cells and tissues and is an important oncogene in HCC. In vitro, LINC01343 knockdown Hep3B and Huh-7 cells showed enhanced apoptosis and inhibited proliferation and migration. An in vivo study further confirmed that LINC01343 knockdown can inhibit tumor growth. In terms of mechanism, LINC01343 plays a role by regulating the LINC01343/miR-526b-5p/ROBO1 axis. LINC01343 directly absorbs miR526b-5p and negatively regulates its expression. ROBO1 was identified as a direct target of miR-526b-5p. ROBO1 knockdown weakened the migration and proliferation of Hep3B and Huh-7 cells. These indicate that LINC01343 may be a potential therapeutic target for HCC [79]. The expression of FAM111A was positively correlated with the m6A level of FAM111A-DT, and the expression of methyltransferase complex, YTHDC1, and KDM3B in HCC tissues. Depletion of FAM111A largely attenuated the roles of m6A­modified FAM111A­DT in HCC. By and large, the m6A­modified FAM111A-DT/YTHDC1/KDM3B/FAM111A regulatory axis promoted HCC growth and represented a candidate therapeutic target for HCC [80]. LINC01468-mediated lipogenesis promotes HCC progression through CUL4A-linked degradation of SHIP2. LINC01468 acts as a driver of HCC progression from NAFLD, highlights the potential of the LINC01468–SHIP2 axis as a therapeutic target for HCC [81]. Studies have found that HOXC-AS3 is highly expressed in HCC and can promote HCC progression by interacting with CDK2. Therefore, targeting HOXC-AS3 is very likely to provide a new strategy for the treatment of HCC and for improving patient prognosis [82]. Mechanistic studies showed that CERS6–AS1 may sponge miR-30b-3p to elevate MDM2, thus promoting the MDM2-mediated ubiquitin-dependent degradation of the p53 tumor suppressor. MDM2 overexpression or miR-30b-3p inhibitors blocked the inhibitory effect of CERS6–AS1 knockdown on proliferation, migration and glycolysis. In summary, the CERS6–AS1/miR-30b-3p/MDM2/p53 signaling axis may play a key role in regulating HCC progression. CERS6–AS1 may exert as a novel biomarker or therapeutic target for HCC [83]. In addition, TLNC1 is a marker of important prognostic value in HCC, which is formed with TPRp53. It exerts its tumorigenic function in the following ways: TLNC1 interacts with TPR to induce TPR-mediated p53 transport from the nucleus to the cytoplasm, thereby inhibiting the transcription of p53 target genes and ultimately promoting the progression of HCC [84]. Moreover, targeting lipogenesis-related lncRNAs have been identified as an effective therapy for some cancers. LINC00958 can promote the expression of hepatoma-derived growth factors, facilitating HCC progression. Encapsulating LINC00958 siRNA into a poly lactic-co-glycolic acid-based nanoplatform has been identified as a therapeutic strategy with satisfactory antitumor efficacy [85].

In summary, lncRNAs such as HClnc1, LINC01343, FAM111A-DT, CERS6–AS1 and TLNC1 significantly affect the progression of HCC by regulating key signal axis or protein function, and are closely related to the prognosis of patients. At the same time, some lncRNAs such as UPF1 and LINC01468–SHIP2 axis were found to have potential therapeutic target value. These studies not only deepen the understanding of the molecular mechanism of HCC, but also provide a theoretical basis and new direction for the development of precise diagnosis and treatment strategies based on lncRNA.

### LncRNAs regulate HCC metabolic reprogramming

LncRNAs have been shown to play an important role in controlling metabolic reprogramming. Emerging studies have shown that lncRNAs control metabolic changes including lipid metabolism reprogramming and play an important role in the progression of HCC. However, how lncRNAs affect tumor cell metabolism remains to be elucidated [86]. As a specific up-regulated lncRNA in HCC, lncRNA HULC can down-regulate miR-9 and relieve the inhibition of PPARα by promoting the demethylation of CpG island in human promoter [71]. Subsequently, activated PPARα further activates ACSL1, thereby promoting long-chain fatty acid metabolism in human Huh7 and HepG2 liver cell lines. In addition, in the liver, the production of triglycerides and cholesterol is also dependent on the role of ACSL1. It is worth noting that cholesterol can also up-regulate HULC expression through a positive feedback mechanism [71]. Therefore, HULC-promoted fatty acid synthesis accelerates the proliferation of HCC cells.

IncRNA-p21 is a hypoxia-responsive lncRNA, which is essential for hypoxia to enhance glycolysis. IncRNA-p21 can regulate the transcriptional activity of HIF-1α by regulating the expression level of HIF-1α under hypoxic conditions, and promote glucose uptake and lactic acid production [87]. By knocking out lncRNA-p21, the activity of LDHA and the expression levels of LDHA and GLUT1 were decreased, indicating that lncRNA-p21 promotes glycolysis under hypoxia. LncRNA RP11-386G11.10 is a novel oncogenic lncRNA, which is closely related to the poor prognosis of HCC [86]. The ZBTB7A-RP11-386G11.10-HNRNPU positive feedback loop promotes the progression of HCC by regulating lipid anabolism. RP11-386G11.10 acts as a competitive endogenous RNA of miR-345-3p to regulate the expression of HNRNPU and its downstream lipogenic enzymes, leading to lipid accumulation in HCC cells and promoting their growth and metastasis [86]. In addition, ZBTB7A is a transcription factor of RP11386G11.10.HNRNPU promotes the expression of ZBTB7A in HCC cells, thereby increasing the transcriptional activity of RP11386G11.10 and forming a positive feedback loop, which ultimately leads to continuous lipid accumulation, growth and metastasis in HCC cells [86]. Using a humanized mouse model, LINC01028 was reported to regulate the expression of multiple genes, such as ADH1C, CYP4A11, and CYP4A22, which were involved in fatty acid oxidation in the liver of humanized mice [88]. LncRNA SLC2A1-DT is up-regulated and activates glycolysis through m6A modification, and promotes HCC progression by stabilizing β-catenin. Studies have shown that SLC2A1-DT binds to YWHAZ. YWHAZ is known to be an oncogene of HCC [89]. At the same time, the high expression of YWHAZ enhanced cell proliferation, metastasis and reduced apoptosis in HCC. YWHAZ overexpression enhances β-catenin-mediated transcription by up-regulating β-catenin levels in the cytoplasm and nucleus [90]. The above studies have shown that YWHAZ binds to β-catenin, resulting in the dissociation of β-catenin from the E3 ubiquitin ligase β-TrCP, which enhances the stability of β-catenin [89].

Table 1 provides a comprehensive overview of these lncRNAs, detailing their specific roles and diagnostic potential in HCC. Therefore, the currently discovered lncRNAs may only constitute part of the potential biomarker spectrum. Future research still needs to be further explored to discover new lncRNAs and further elucidate their mechanisms in the occurrence and development of HCC.

**Table 1:** Summary of the cellular functions of lncRNAs in tumorigenesis of HCC

LncRNA	Role	Key factors	Outcome	Ref.
linc01134	Oncogenic	SSRP1	Proliferation ↑	[23]
LncRNA NEAT1	Oncogenic	Bcl-2/EGFR	Proliferation ↑, Invasion ↑	[34]
lncRNA_ DSCR8	Dual	miR-22-3p	Metastasis modulation	[35]
lncRNA PNUTS	Oncogenic	ZEB1	Proliferation ↑, Metastasis ↑	[91]
lncRNA02273	Suppressor	Hep3B/MHCC97	Apoptosis ↑	[36]
lncRNA HULC	Oncogenic	HOTAIR	Proliferation ↑, Invasion ↑, Metastasis ↑	[91]
lncRNA KCNQ1OT1	Oncogenic	S1PR1	Tumorigenesis ↑	[37]
FAT1	Suppressor	miR-223-3p	Proliferation ↓, Metastasis ↓, Invasion ↓, EMT↓	[41]
CYTOR	Suppressor	HepG2/SMMC-7721	Apoptosis ↑	[45]
SNHG4	Oncogenic	SNHG4/miR-2115p/CREB5	Stage ↑, Tumor growth ↑	[47]
DACT3–AS1	Oncogenic	HDAC2/FOXA3	Metastasis ↑	[49]
H19	Oncogenic	CDC42/PAK1	Proliferation ↑, Invasion ↑, Metastasis ↑	[50]
HOTTIP	Oncogenic	miRNA-125b	Invasion ↑, Metastasis ↑	[51]
lncRNA CRNDE	Oncogenic	Wnt/β-catenin	Tumor growth ↑, Metastasis ↑	[52]
ZFPM2–AS1	Oncogenic	miR-3065-5p/XRCC4	DNA damage repair ↑, Proliferation ↑	[63]
lncRNA AL133467.2	Oncogenic	ZCCHC4	Apoptosis ↓, Chemosensitivity *↑*	[64]
lncRNA KCNQ1OT1	Suppressor	miR-146a-5p	Apoptosis *↑*, Radiosensitivity ↓	[71]
BCLF1	Oncogenic	NEAT1	Proliferation ↑, Invasion ↑	[70]
HULC	Oncogenic	LC3II	Proliferation ↑, Tumor growth ↑	[75]
Linc01343	Oncogenic	LINC01343/miR-526b-5p/ROBO1	Proliferation ↑, Invasion ↑, Metastasis ↑	[79]
LINC01468	Oncogenic	LINC01468–SHIP2	Tumor growth ↑, Metastasis ↑	[81]
HOXC-AS3	Oncogenic	CDK2	Stage *↑*	[82]
CERS6–AS1	Oncogenic	CERS6–AS1/miR-30b-3p/MDM2/p53	Tumorigenesis *↑*	[83]
TLNC1	Oncogenic	TPR	Tumorigenesis *↑*, Stage *↑*	[84]
LncRNA RP11-386G11.10	Oncogenic	ZBTB7A-RP11-386G11.10-HNRNPU	Tumorigenesis *↑*, Stage *↑*	[86]
HNRNPU	Oncogenic	ZBTB7A	Proliferation ↑, Tumor growth ↑, Metastasis ↑	[86]
YWHAZ	Oncogenic	SLC2A1-DT	Proliferation ↑, Metastasis ↑, Apoptosis ↓	[89]

↑ means promote/increase, ↓ means inhibit/decrease

## Mechanisms of lncRNA-mediated immune escape

### Regulation of immune checkpoints

For many years, the primary treatment for advanced HCC has been systemic therapy, and tyrosine kinase inhibitors (TKIs), represented by sorafenib and lenvatinib, have been the first-line treatment for advanced HCC, bringing hope to many HCC patients [92]. However, immunotherapies such as immune-checkpoint inhibitors (ICIs) are revolutionizing the management of cancer. Antibodies that obstruct these immune checkpoints can potentially activate tumor-specific T cells, thereby amplifying antitumor activity [93]. For advanced HCC, the combination of atezolizumab and bevacizumab is currently positioned as the first-line treatment for patients with advanced HCC [94].

Research indicates that specific lncRNAs can influence immune cell function in the TME by upregulating or down regulating the expression of immune checkpoint molecules. A recent report showed that a single CD-155/TIGIT (T cell immuno77receptor with Ig and ITIM domains) or PD-1/PD-L1 (Programmed death-1/Programmed death Ligand 1) blockade has limited anti-tumor effects [95]. TIGIT blockade not only targets the anti-tumor effector T cell response, but also targets the induction of NK cell killing ability and regulatory T cell inhibition ability. The lncRNAs expected to target PD-L1 and CD-155 transcripts were CCAT-1, H19, and MALAT-1 [95]. Through experiments, knocking down lncRNAs-CCAT-1, MALAT-1 or H19-significantly reduced the co-expression of PD-L1 and CD155. This highlights the immunoregulatory role of CCAT-1, H19 [96] and MALAT-1 in HCC. In addition, hypoxia can lead to immune escape of HCC and promote the expression of PD-L1 through HIF-1α-induced MIR155 HG (the MIR155 host gene). MIR155 HG regulates the expression of PD-L1 by binding to ILF3 (interleukin-enhancing binding factor 3) to regulate the stability of HIF-1α (hypoxia-inducing factor 1α). Therefore, the hypoxia-induced HIF-1α/MIR155HG positive feedback loop provides a new perspective for the immune status of HCC and lays a foundation for the development of more effective immunotherapy strategies [97].

All things considered, the treatment of advanced HCC is shifting from traditional tyrosine kinase inhibitors to immunotherapy such as ICIs. For example, atezolizumab combined with bevacizumab has become the preferred regimen. Specific lncRNAs, such as CCAT-1, H19 and MALAT-1, affect the tumor microenvironment by regulating the expression of immune checkpoint molecules, revealing new immunotherapy targets. In addition, the hypoxia-induced HIF-1α/MIR155 HG loop promotes PD-L1 expression, leading to HCC immune escape, providing a new perspective for the development of more effective immunotherapy strategies. Here are some immunotherapy regimens for HCC (Table 2).

**Table 2:** Monoclonal antibody therapy in different clinical trials

Treatment	Immunotherapeutic agent	Phase	Clinical trials. gov ID	Ref.
First-line treatment	Atezolizumab plus bevacizumab	III	NCT03434379	[98]
	Sintilimab plus a bevacizumab biosimilar (IBI305)	III	NCT03794440	[99]
	Tremelimumab plus durvalumab	III	NCT03298451	[100]
Second-line treatment	Pembrolizumab	III	NCT02702401	[101]
	Nivolumab	I/II	NCT01658878	[102]
	Tislelizumab	II	NCT03419897	[103]
	Sintilimab plus IBI310 (anti-CTLA4 mAb)	Ib	NCT04401813	[104]

### Apoptosis and immune escape

Apoptosis is a vital mechanism for maintaining homeostasis in organisms, and tumor cells escape immunity by regulating apoptotic mechanisms. Certain lncRNAs enhance tumor cell survival by suppressing genes linked to apoptosis [93]. For example, lnc-AIFM2–1, as the ceRNA (endogenous RNA) of miR-330-3p, can inhibit the metazyme activity of lnc-AIFM2–1. At the same time, the competition between lnc-AIFM2–1 and miR-330-3p can inhibit the expression of CD244 (signaling lymphocyte activation molecule family member 4) on CD8^+^ T cells, thereby helping tumor cells escape immune clearance [105].

### Holistic view of lncRNA-TME interactions

LncRNAs in the tumor microenvironment (TME) are involved in regulating immune escape and have intricate interactions with other cell types. HCC, in particular, features a highly intricate TME. For instance, lncRNA MEG3 exhibits a negative correlation with macrophage polarization regulator 1 (CSF-1). It regulates TME by affecting TAM through CSF-1, thereby affecting the balance of Th1/Th2 cells and changing the expression of PD-1/PD-L1s. Overexpression of MEG3 can lead to a significant reduction in tumor growth, decreased expression of PD-1/PD-L1s in macrophages, and enhanced Th1 response. Conversely, knockdown of MEG3 promotes tumor progression by upregulating PD-1/PD-L1 expression and shifting immunity toward a Th2-skewed response [106].

## Potential of lncRNAs as therapeutic targets

### Therapeutic strategies for lncRNA targeting

LncRNAs regulate gene expression and protein synthesis through diverse molecular mechanisms. Physiologically, aberrant expression of lncRNAs can promote oncogenic pathways and suppress tumor suppressor genes, thereby contributing to hepatocarcinogenesis, proliferation, cell growth, invasion and metastasis [51]. Studies have shown that specific inhibition of specific lncRNAs can effectively inhibit the proliferation and metastasis of tumor cells. For example, lncRNA MIR31HG sponges oncogenic miR-575, thereby preventing inhibition of Suppression of Tumorigenicity 7-Like (ST7L) and inducing tumor repression [107]. Moreover, recent studies have shown that CASC2c tends to be expressed at low levels in HCC tissues and cells, and CASC2c expression strongly prevents proliferation and reduces invasiveness, but induces apoptosis in HCC [108]. Functionally, CASC2c serves as a key regulator of the ERK1/2 and Wnt/β-catenin signaling pathways. Upregulation of CASC2c suppresses the activation of these pathways, leading to increased apoptosis and decreased invasion and proliferation [108]. Additionally, the lncRNA AC115619 has been found to encode a micropeptide named AC115619-aa, which is down-regulated in human HCC and exhibits tumor-suppressive properties [109]. This micropeptide interacts with WTAP to disrupt the assembly of the N6-methyladenosine (m6A) methyltransferase complex. Consequently, it reduces global m6A levels in HCC cells and modulates the expression of tumor-associated proteins SOCS2 and ATG14 [110].

### Challenges and future perspectives in clinical applications

LncRNAs are key mediators with a wide range of pathophysiological functions, but their roles in human HCC remain largely unknown and are slowly being explored. Research on lncRNAs has opened new avenues for understanding the mechanisms of various physiological and pathological processes. Compelling evidence has revealed an intrinsic link between lncRNAs and lipid metabolism, demonstrating that lncRNAs-induced disruption of lipid metabolism and signaling contribute to the development of multiple cancers and some other diseases, including obesity, fatty liver disease, and cardiovascular disease [111]. Despite the abundance of lncRNA loci identified, their functions in metabolic regulation are still poorly understood. deeper insight into the roles and mechanisms of lncRNAs could offer novel perspectives for studying lipid metabolism and signaling, and developing lncRNA-based therapeutics may represent a promising strategy for treating lipid metabolism-related diseases [111].

HCC is characterized by high heterogeneity, with diverse etiologies leading to distinct driver mutations and unique tumor immune microenvironments. Current therapeutic options, including immune checkpoint inhibitors and combinations, have achieved limited objective response rates for the majority of patients. Thus, a precision medicine approach is needed to tailor specific treatment options for molecular subsets of HCC patients [112].

## Conclusion and perspective

This article systematically reviews the research progress of lncRNA in HCC, emphasizing its key role in disease development, diagnostic evaluation and therapeutic intervention. With the in-depth exploration of the regulatory mechanism of lncRNAs, more and more studies have revealed their multiple functions in gene expression regulation, signal pathway influence, metabolic reprogramming, and immune microenvironment regulation. LncRNAs have become an important research direction for precise diagnosis and treatment of HCC.

In the future, with the continuous development of high-throughput sequencing technology and bioinformatics analysis methods, more novel lncRNAs with biological significance and clinical value are expected to be discovered and their molecular mechanisms in HCC will be further clarified. In addition, how to effectively transform these lncRNAs into clinically available biomarkers or therapeutic targets will be the focus of research. For example, by developing efficient lncRNA detection technology to improve its detection sensitivity and specificity in blood or other body fluid samples, it is helpful to achieve early non-invasive diagnosis and dynamic monitoring of HCC. At the same time, lncRNA-based targeted therapy strategies, such as intervention using siRNA or antisense oligonucleotide (ASO), will also provide more personalized and safe treatment options for HCC patients.

More importantly, with the continuous advancement of research and the continuous optimization of technical platforms, lncRNAs are expected to become a core component of the HCC diagnosis and treatment system in the future, bringing a truly revolutionary breakthrough for HCC patients.

## Data Availability

No datasets were generated or analysed during the current study.
